# Identification of the Allergenic Ingredients in Reduning Injection by Ultrafiltration and High-Performance Liquid Chromatography

**DOI:** 10.1155/2016/4895672

**Published:** 2016-04-10

**Authors:** Fang Wang, Cun-yu Li, Yun-feng Zheng, Hong-yang Li, Wei Xiao, Guo-ping Peng

**Affiliations:** ^1^College of Pharmacy, Nanjing University of Chinese Medicine, Nanjing 210023, China; ^2^Collaborative Innovation Center of Chinese Medicinal Resources Industrialization, Jiangsu, China; ^3^Jiangsu Kanion Pharmaceutical Co., Ltd., China

## Abstract

*Reduning* injection is a traditional Chinese medicine injection which has multiple functions such as clearing heat, dispelling wind, and detoxification. Although* Reduning* injection was widely utilized, reports of its allergenicity emerged one after another. However, there is little research on its allergenic substances. The aim of this study is to evaluate the sensitization of* Reduning* injection and explore the underlying cause of the anaphylactic reaction. The main ingredients in* Reduning* injection were analyzed before and after ultrafiltration. Ultrafiltrate* Reduning* injection, unfiltered* Reduning* injection, egg albumin, Tween-80, and nine effective components in* Reduning* injection were utilized to sensitize guinea pigs. The serum 5-hydroxytryptamine level was used to assess the sensitization effect of* Reduning* injection. We found a significant decrease in Tween-80 content comparing to other components in the injection after ultrafiltration. Unfiltered* Reduning* injection, Tween-80, chlorogenic acid, and cryptochlorogenin acid caused remarkable anaphylactoid reaction on guinea pigs while ultrafiltration* Reduning* resulted in a significantly lower degree of sensitization. Our results suggest that ultrafiltration could significantly reduce the sensitization of* Reduning* injection, which is likely due to the decrease of Tween-80. We also conjectured that the form of chlorogenic acid and cryptochlorogenin acid within the complex solution mixture may also affect the sensitizing effect.

## 1. Introduction

*Reduning* injection is a traditional Chinese medicine (TCM) injection refined from three Chinese herbal medicines, namely, honeysuckle, gardenia, and abrotani herba, and formulated for injection [1, 2]. This injection has multiple functions such as clearing heat, dispelling wind, and detoxification [3]. It is clinically used in the treatment of hyperpyrexia, slight aversion to cold, head and body pain, cough, yellow sputum, and other symptoms caused by respiratory tract infection (with external wind heat syndrome) [4, 5]. From the time* Reduning* injection was listed as a clinical treatment, it has been widely utilized with good clinical efficacy. However, there are also a few reports of severe anaphylaxis during the clinical application [6]. It is known that* Reduning* injection mainly contains Tween-80, chlorogenic acid, geniposide, caffeic acid, cryptochlorogenin acid, isochlorogenic acid (A, B, and C), neochlorogenic acid, and secoxyloganin [7–10]. Previous studies have shown that some components in TCM injections might be the cause of the allergic reaction and anaphylactoid reaction [11–13]. However, the specific compound responsible for the allergic reaction is still not clear.

Ultrafiltration is a separation technique in which a porous membrane is used as filtering medium and the molecules are separated according to their size [14]. After ultrafiltration, macromolecules such as pyrogen, microbes, proteins, pigments, resin, and tannin are excluded [15]. In this study, we used ultrafiltration to separate the compounds in* Reduning* injection to explore the cause of anaphylaxis. Previous studies have identified some key cytokines are the mediator of antibody-mediated systemic anaphylaxis [16–18] and our studies had shown that 5-hydroxytryptamine (5-HT) can be detected rapidly by high-performance liquid chromatography (HPLC) and can be used as an index for anaphylaxis [19]. In our study, guinea pig plasma 5-HT level was measured to determine the sensitization of* Reduning* injection before and after ultrafiltration. Furthermore, the components in the injection were also analyzed to determine the main allergens in* Reduning* injection.

## 2. Materials and Methods

### 2.1. Animals

A total of 140 male guinea pigs were purchased from Nanjing Qinglongshan Animal Breeding Center. All animals were housed at 22°C and 55% ±5% relative humidity. All experiments were carried out according to the guidelines of the Animal Care Committee of Nanjing University of Chinese Medicine.

### 2.2. Reagents and Materials

Egg albumin and 5-HT were purchased from Sigma (St. Louis, USA). Chloral hydrate was purchased from Sinopharm Chemical Reagent Co., Ltd. (Shanghai, China). Heparin sodium injection was purchased from Jiangsu Wangbang Pharmaceutical Co., Ltd. (Jiangsu, China). Heptanesulfonate sodium was purchased from J&K Chemical Ltd. (Shanghai, China). Sodium dihydrogen phosphate was purchased from Shantou Xilong Chemical Industry Factory Co., Ltd. (Guangdong, China). Ethylene Diamine Tetraacetic Acid (EDTA) was purchased from Shanghai Fuqi Industrial & Trading Co., Ltd. (Shanghai, China). Sodium chloride injection was purchased from Anhui Fengyuan Pharmaceutical Co., Ltd. (Anhui, China).

*Reduning* injection was purchased from Jiangsu Kangyuan Pharmaceutical Co., Ltd. (Jiangsu, China), the batch numbers are 120211, 120209, and 120703. Tween-80 was purchased from Sigma (St. Louis, USA), the batch number is 018K00941. Chlorogenic acid was purchased from China Institute of pharmaceutical and biological products (Beijing, China); the batch number is 0753-9909. Geniposide was purchased from National Institute for Food and Drug Control (Beijing, China); the batch number is 110885-200102. Neochlorogenic acid and cryptochlorogenin acid were purchased from Shanghai Yuanye Technology Co., Ltd. (Shanghai, China); the batch numbers are PA0819RA13 and ZF0226BA14. Isochlorogenic acid A, isochlorogenic acid B, isochlorogenic acid C, and secoxyloganin reference substance were produced in-house and possessed a purity above 98% as tested by chromatography. The ultrafiltration column was purchased from Millipore Corporation (Massachusetts, USA).

### 2.3. Solutions Preparation

Egg albumin solution was dissolved in saline to a concentration of 5.0 mg/mL. Tween-80 was prepared to 1% v/v by dissolving 1 mL of Tween-80 in saline.

Standard solutions of chlorogenic acid, neochlorogenic acid, cryptochlorogenin acid, geniposide, caffeic acid, isochlorogenic acid A, isochlorogenic acid B, isochlorogenic acid C, and secoxyloganin were prepared in saline, and their concentrations were 6.0 mg/mL, 3.7 mg/mL, 3.3 mg/mL, 7.0 mg/mL, 0.3 mg/mL, 0.3 mg/mL, 0.6 mg/mL, 0.5 mg/mL, and 1.0 mg/mL, respectively. The concentrations are equal to their respective concentrations in the* Reduning* injection.

After equilibration with two column volumes of* Reduning* injection, the ultrafiltration column with a molecular weight cut-off of 2,000 Da was used to filter the* Reduning* injection.

### 2.4. Detection of Major Components in* Reduning* Injection before and after Ultrafiltration

#### 2.4.1. Simultaneous Detection of Chlorogenic Acid, Neochlorogenic Acid, Cryptochlorogenin Acid, Geniposide, Caffeic Acid, Isochlorogenic Acid A, Isochlorogenic Acid B, Isochlorogenic Acid C, and Secoxyloganin

Waters e2695 liquid chromatograph equipped with a Waters 2489 UV/visible light detector (Massachusetts, USA) was utilized for detection. The ODS-C18 (4.6 mm × 150 mm, 5 *μ*m) column was used with a flow rate of 1.0 mL/min at a column temperature of 30°C. The mobile phases were methyl alcohol and 0.1% formic acid-water. The elution gradient program is shown in Table 1. The detection wavelength was 238 nm.

#### 2.4.2. Detection of Tween-80

Waters e2695 liquid chromatograph equipped with an Alltech evaporative light scattering detection 2000 detector (Kentucky, USA) was used for detection. A TSK-GEL G2000 SWXL (7.8 mm × 300 mm, 5 *μ*m) column was used for separation. The mobile phases were 5.0 mmol·L^−1^ ammonium acetate : acetonitrile (90 : 10), pH = 4.0, and column temperature was 30°C. Flow rate was 0.6 mL·min^−1^ and the injection volume was 10 *μ*L. As for the ELSD condition, the drift tube temperature was set at 110°C and nitrogen flow rate was set at 2.3 L·min^−1^.

### 2.5. Calculation of Componential Transmittance

The percentage of retention of each component following ultrafiltration was calculated according to

(1) $$\begin{array}{c} R=\frac{C_{f}}{C_{s}}\times 100\%, \end{array}$$

where *R* is the percentage of retention of the component, *C*_*f*_ is the concentration of components in filtrate (mg/mL), and *C*_*s*_ is the concentration of components in stock solution (mg/mL).

### 2.6. Animal Experiments

A total of 140 male guinea pigs with bodyweight of 300 ± 50 g were randomly divided into 14 groups and used and a modified anaphylaxis examination method was established according to the Guidelines for Safety Tests on TCM Injections in the appendix of Chinese Pharmacopoeia 2010. Each guinea pig was subcutaneously injected with 0.2 mL solution of the test solution (saline, egg albumin, ultrafiltered* Reduning *injection, unfiltered* Reduning* injection, Tween-80, chlorogenic acid, geniposide, caffeic acid, neochlorogenic acid, cryptochlorogenin acid, isochlorogenic acid A, isochlorogenic acid B, isochlorogenic acid C, and secoxyloganin) once every other day (three times in total) to test for sensitization. On the 16th day after the first injection, 10% chloral hydrate (0.3 mL/100 g) was used to anesthetize the guinea pigs. Animals were stimulated by jugular vein injection with 0.5 mL solution of each test solutions, respectively. Blood (1 mL) was extracted from the carotid artery and collected in a heparin anticoagulation tube before (sample A) and after (sample B) the stimulation, and the samples after the stimulation were collected at 30 min. Blood samples were centrifuged at 4,000 rpm for 5 min and the plasma was stored at −80°C.

### 2.7. Detection of 5-HT

Plasma sample (100 *μ*L) was thawed in the dark and mixed by vortex with an equal amount of methanol. The mixture was centrifuged at 3,000 rpm for 10 min. After freezing for 30 min, the solution was centrifuged at 1,000 rpm for 10 min and 20 *μ*L supernatant was used for detection.

Waters 510 liquid chromatograph was used with a Waters 2465 electrochemical detector (Massachusetts, USA) and EC 2000 chromatographic work station (Dalian, China). Hedera ODS-2 chromatographic column (150 mm × 4.6 mm, 5 *μ*m, Jiangsu Hanbang Science & Technology Co., Ltd.) was utilized at a column temperature of 25°C. Mobile phase was 25 mmol/L sodium dihydrogen phosphate (containing 0.5 mmol/L EDTA and 3 mmol/L sodium heptanesulfonate, pH 4.6) mixed with acetonitrile at a volume ratio of 85 : 15. The flow velocity was 0.8 mL/min. ISAAC (in situ silver/silver chloride) was used as the reference electrode. The detection potential was 0.6 V and the injection volume was 20 *μ*L. The amount variation rate of 5-HT was calculated according to the following equation:

(2) $$\begin{array}{c} \text{Sample }\left(\;\%\;\right)=\frac{\left(\text{value B}-\text{value A}\right)}{\text{value A}}\times 100\%, \end{array}$$

where value B is the amount of 5-HT in sample B and value A is the amount of 5-HT in sample A.

### 2.8. Statistical Analysis

Data were reported as mean ± SD for each group. All statistical analyses were performed using PRISM version 5.0 (GraphPad). Differences with *P* value of less than 0.5 were considered statistically significant.

## 3. Results

### 3.1. Calibration Curves of the Investigated Compounds

The mixture of standard solutions, Tween-80, and 5-HT reference solution were diluted to different concentrations. We assigned the compound concentration as the horizontal axis (*X*) and the peak area as the vertical axis (*Y*) and performed a linear regression to obtain the regression equations of each component. The results are shown in Table 2. The results showed that for all the standard solutions, there is a good linear relationship between the compound concentration and the area under the peak within a wide range.

The method precision was evaluated by intraday and interday variability. The intraday variability was performed by injection of the same sample six times in the same day. The interday variability was evaluated on two successive days using the same sample. The RSD values are summarized in Table 3. From the results, RSDs for intraday and interday precisions did not exceed 3%. To confirm the repeatability of the method, six independently prepared solutions from the same sample were analyzed. The stabilities of the sample solutions were analyzed at 0, 2, 4, 8, 12, and 24 h at room temperature. It was found that the sample solutions were stable within 24 h. Precision, repeatability, and stability of nine compounds (*n* = 6), the RSD values, are summarized in Table 3.

The recovery was evaluated by adding standards into the sample. The mixture was extracted and analyzed by using the above method. Six replicates were performed for the determination. The RSD values are summarized in Table 4.

**Figure 1:**
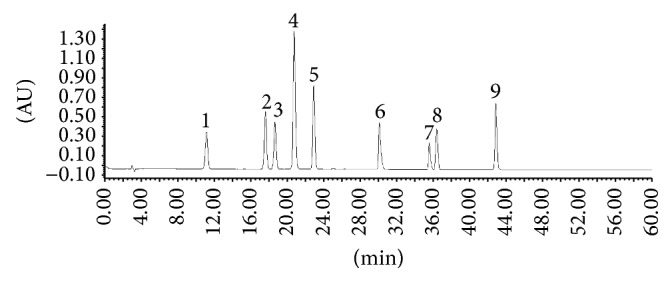
HPLC chromatograms of standard solution of nine compounds: (1) neochlorogenic acid; (2) chlorogenic acid; (3) cryptochlorogenin acid; (4) caffeic acid; (5) geniposide; (6) secoxyloganin; (7) isochlorogenic acid B; (8) isochlorogenic acid A; (9) isochlorogenic acid C.

**Figure 2:**
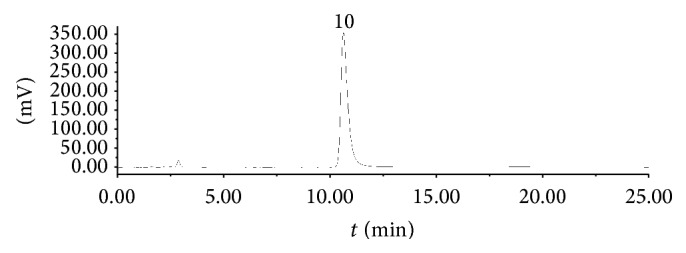
HPLC chromatograms of Tween-80.

**Figure 3:**
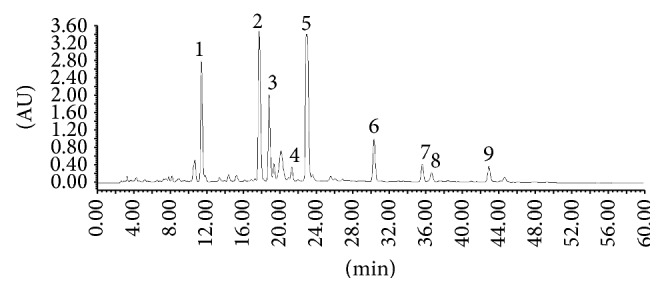
The chromatogram of* Reduning *injection: (1) neochlorogenic acid; (2) chlorogenic acid; (3) cryptochlorogenin acid; (4) caffeic acid; (5) geniposide; (6) secoxyloganin; (7) isochlorogenic acid B; (8) isochlorogenic acid A; (9) isochlorogenic acid C.

HPLC chromatogram of the standard solution mixture was shown in Figure 1, the chromatogram of Tween-80 reference solution was shown in Figure 2. The chromatogram of* Reduning* injection was shown in Figure 3.

### 3.2. Percentage of Retention of Major Components after Ultrafiltration

An ultrafiltration membrane was used to filter the* Reduning* injection. The permeation of molecular through the membranes was not entirely related to the monomolecular weight of composition, but to the molecular's existence formed (monomolecular, low polymolecular, and high polymolecular) in the solution. The concentrations and percentage of retention of the major components contained in the injection both before and after ultrafiltration are shown in Table 5. The percentage of retention of chlorogenic acid, geniposide, caffeic acid, neochlorogenic acid, and cryptochlorogenin acid is all greater than 90%, while the percentage of retention of secoxyloganin, isochlorogenic acid A, isochlorogenic acid B, and isochlorogenic acid C is all greater than 70%. The percentage of retention of Tween-80 is 20%.

### 3.3. Plasma 5-HT in Guinea Pigs

**Figure 4:**
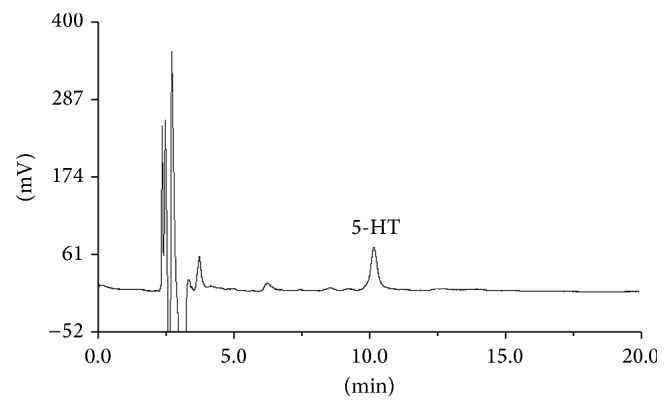
The chromatogram of 5-HT.

**Figure 5:**
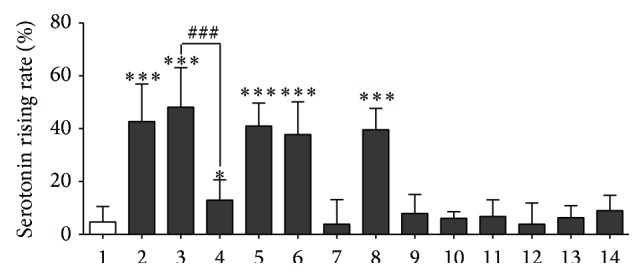
Increasing rate of 5-HT in guinea pig plasma; (1) saline; (2) egg albumin; (3)* Reduning* injection; (4) filtered* Reduning* injection; (5) Tween-80; (6) chlorogenic acid; (7) neochlorogenic acid; (8) cyclohexanecarboxylic acid; (9) gardenoside; (10) caffeic acid; (11) isochlorogenic acid A; (12) isochlorogenic acid B; (13) isochlorogenic acid C; (14) secoxyloganin. Values are expressed as mean ± SD; *n* = 10 in each group. ^*∗*^ *P* < 0.05, and ^*∗∗∗*^ *P* < 0.001 versus saline. ^###^ *P* < 0.001 versus filtered* Reduning* injection.

The chromatogram of 5-HT was shown in Figure 4. After the guinea pigs were sensitized to saline for 30 min by injection, the average increasing rate of plasma 5-HT was 4.46%. The average increasing rate in plasma 5-HT content following sensitization to egg albumin, unfiltered* Reduning*, Tween-80, chlorogenic acid, and cryptochlorogenin acid were significant, ranging from 37.75% to 48.11%. In contrast, then, the guinea pigs were sensitized to* Reduning* ultrafiltrate; the average increasing rate in plasma 5-HT was reduced significantly compared to unfiltered* Reduning*. The average increase rate in plasma 5-HT following sensitization to neochlorogenic acid, geniposide, caffeic acid, secoxyloganin, and all three isochlorogenic acid isomers were all below 10%. Each group of data and sodium chloride injection group were conducted with one-way ANOVA, the results of which were shown in Figure 5.

## 4. Discussion

Allergic reaction is the most common adverse reaction of herbal injection which could lead to life-threatening health problems. Commonly used methods such as murine passive cutaneous anaphylaxis and measurement of the antibodies were expensive and time-consuming work. Previous studies have identified two types of antibody-mediated systemic anaphylaxis; one is mediated by IgE and the other mediated by IgG [20–23]. Mast cells are the major effector cells of IgE mediated in allergic reaction and play a key role in allergic reaction diseases [24]; when anaphylactic reaction occurs, mast cells and basophils would degranulate and release *β*-hexosaminidase, histamine, serotonin, or other cytokines [25–28]. In this study, a simple and rapid method was discussed based on the measurement of serotonin with HPLC technique for predicting allergenic reaction of all the analytes.

Previous studies have reported that the chlorogenic acid in honeysuckle may cause anaphylaxis [29, 30]. In present study, we investigated sensitization to the major phenolic acid components in* Reduning* injection in guinea pigs. Our results indicate that chlorogenic acid causes sensitization, which is consistent with previous reports. At the same time, we found that cryptochlorogenin acid also could cause anaphylactoid reaction even stronger than chlorogenic acid. Other components, such as caffeic acid, neochlorogenic acid, isochlorogenic acid A, isochlorogenic acid B, isochlorogenic acid C, and secoxyloganin do not induce obvious sensitization. However, concentration of chlorogenic acid and cryptochlorogenin acid in filtered* Reduning* is similar to its concentration before ultrafiltration, yet we detected obvious sensitization in guinea pigs using unfiltered* Reduning* while ultrafiltered* Reduning* exhibited no significant sensitization, suggesting that there is a remarkable difference between sensitizations to chlorogenic acid and cryptochlorogenin acid as an isolated compound and as a component in a mixed solution. The difference can likely be attributed to the form of chlorogenic acid and cryptochlorogenin acid within the complex solution mixture. In herbal medicine solution, the compounds exist in many forms such as ionic, molecular, and associated.

Tween-80 is a nonionic detergent and is mostly used in Chinese medicine injections to assist in solubilization [31, 32]. However, adverse reactions of Tween-80 have been observed with time. There are several Chinese medicine injections in clinical use that contain Tween-80, such as* Yuxingcao* injection and* Xiangdan* injection, both of which have been reported to have serious adverse reactions [13]. The percentage of retention of Tween-80 in ultrafiltered* Reduning* injection was low, resulting in decreased Tween-80 and decreased sensitization, which suggests that any observed sensitization to* Reduning* injection was likely due to the presence of Tween-80.

## 5. Conclusions

In this study, a simple HPLC technique was successfully used for predicting allergenic reaction of all the analytes in* Reduning* injection before and after ultrafiltration and we suggested that Tween-80 might be the cause of anaphylaxis. Additionally, the form of chlorogenic acid and cryptochlorogenin acid within the complex solution mixture may also affect the sensitizing effect of them. Our findings might contribute to the safety evaluation of* Reduning* injection in clinical medication.

## Figures and Tables

**Table 1:** Elution gradient program.

Time/min	Methyl alcohol%	0.1% formic acid-water
0	12	88
20	30	70
60	45	55

**Table 2:** Regression equation, linear range, and correlation coefficient of investigated compounds.

Serial number	Component name	Regression equation	Correlation coefficient (*r*)	Linear range (mg/mL)
1	Neochlorogenic acid	*Y* = 13.223*X* − 6.236	0.9989	0.05~5.05
2	Chlorogenic acid	*Y* = 10.758*X* + 20.156	0.9998	0.04~10.00
3	Cryptochlorogenin acid	*Y* = 10.002*X* + 5.3598	0.9991	0.05~6.12
4	Caffeic acid	*Y* = 20.339*X* + 10.856	0.9995	0.01~4.22
5	Geniposide	*Y* = 5.673*X* + 14.982	0.9993	0.05~10.06
6	Secoxyloganin	*Y* = 12.912*X* − 5.32	0.9992	0.04~5.22
7	Isochlorogenic acid B	*Y* = 14.008*X* + 20.703	0.9987	0.01~3.00
8	Isochlorogenic acid A	*Y* = 16.612*X* + 16.356	0.9995	0.01~3.10
9	Isochlorogenic acid C	*Y* = 16.103*X* + 35.311	0.9993	0.01~4.00
10	Tween-80	*Y* = 1.5269*X* + 7.5201	0.9988	0.50~4.23
11	5-HT	*Y* = 0.5468*X* − 3.5868	0.9989	0.03 × 10^−3^ ~3.00 × 10^−3^

**Table 3:** Precision, repeatability, and stability of nine compounds (*n* = 6).

Serial number	Component name	Precision (*n* = 6)		Repeatability (*n* = 6) RSD (%)	Stability (*n* = 6) RSD (%)
		Intraday RSD (%)	Interday RSD (%)		
1	Neochlorogenic acid	1.32	1.46	2.01	1.56
2	Chlorogenic acid	1.78	2.74	2.39	2.44
3	Cryptochlorogenin acid	1.42	2.03	2.72	2.75
4	Caffeic acid	1.09	1.62	2.47	2.05
5	Geniposide	2.07	1.98	1.59	1.46
6	Secoxyloganin	2.01	2.41	2.84	2.26
7	Isochlorogenic acid B	1.37	1.93	1.23	1.74
8	Isochlorogenic acid A	1.96	2.34	2.68	1.35
9	Isochlorogenic acid C	1.79	2.63	1.86	1.90
10	Tween-80	2.67	2.83	2.40	2.58
11	5-HT	1.39	1.57	1.82	1.66

**Table 4:** Recovery of the compounds in *Reduning* injection.

Serial number	Compounds	Contained (mg/mL)	Added (mg/mL)	Found mean (mg/mL)	Recovery mean (%)	RSD (%) *n* = 6
1	Neochlorogenic acid	3.68	3.56	7.09	104.39	2.36
2	Chlorogenic acid	5.83	5.93	11.98	96.42	1.87
3	Cryptochlorogenin acid	3.30	3.24	6.49	101.56	1.94
4	Caffeic acid	0.34	0.34	0.67	103.03	2.50
5	Geniposide	8.95	8.79	17.93	97.88	1.69
6	Secoxyloganin	1.01	0.98	2.04	95.14	2.15
7	Isochlorogenic acid B	0.62	0.59	1.19	103.50	2.36
8	Isochlorogenic acid A	0.32	0.32	0.65	96.96	2.13
9	Isochlorogenic acid C	0.52	0.50	1.03	98.03	1.98
10	Tween-80	2.20	2.00	4.25	97.56	2.69
11	5-HT	0.091 × 10^−3^	0.092 × 10^−3^	0.186 × 10^−3^	96.84	2.87

**Table 5:** Concentration and percentage of retention of each component before and after ultrafiltration.

Serial number	Name	Before ultrafiltration (mg/mL)	After ultrafiltration (mg/mL)	Percentage of retention (%)	RSD (%) *n* = 3
1	Neochlorogenic acid	3.71	3.42	92.18^*∗∗*^	1.79
2	Chlorogenic acid	6.12	5.46	89.21^*∗∗*^	2.25
3	Cryptochlorogenin acid	3.32	3.00	90.36^*∗∗*^	2.12
4	Caffeic acid	0.31	0.29	93.54^*∗∗*^	2.10
5	Geniposide	9.10	8.90	97.80^*∗∗*^	1.63
6	Secoxyloganin	1.02	0.68	73.67^*∗∗*^	2.13
7	Isochlorogenic acid B	0.60	0.44	73.33^*∗∗*^	1.98
8	Isochlorogenic acid A	0.33	0.25	75.76^*∗∗*^	2.43
9	Isochlorogenic acid C	0.51	0.36	70.58^*∗∗*^	1.59
10	Tween-80	2.00	0.40	20.00	2.58

Values are expressed as mean ± SD; *n* = 3 in each group. ^*∗∗*^ *P* < 0.01 versus Tween-80 group.
